# Female Field Crickets Incur Increased Parasitism Risk When Near Preferred Song

**DOI:** 10.1371/journal.pone.0009592

**Published:** 2010-03-09

**Authors:** Cassandra M. Martin, William E. Wagner

**Affiliations:** School of Biological Sciences, University of Nebraska-Lincoln, Lincoln, Nebraska, United States of America; University of Exeter, United Kingdom

## Abstract

Female animals often prefer males with conspicuous traits because these males provide direct or indirect benefits. Conspicuous male traits, however, can attract predators. This not only increases the risk of predation for conspicuous males but also for the females that prefer them. In the variable field cricket, *Gryllus lineaticep*s, males that produce preferred song types provide females with greater material benefits, but they are also more likely to attract lethal parasitoid flies. First, we conducted a field experiment that tested the hypothesis that females have a greater risk of fly parasitism when in association with preferred high chirp rate males. Females were nearly twice as likely to be parasitized when caged with high chirp rate song than when caged with low chirp rate song. Females may thus be forced to trade off the quality of the benefits they receive from mating with preferred males and the risk of being killed by a predator when near these males. Second, we assessed female parasitism rates in a natural population. Up to 6% of the females were parasitized in field samples. Because the females we collected could have become parasitized had they not been collected, this provides a minimum estimate of the female parasitism rate in the field. In a laboratory study, we found no difference in the proportion of time parasitized and unparasitized females spent hiding under shelters; thus, differences in activity patterns do not appear to have biased our estimate of female parasitism rates. Overall, our results suggest that female association costs have the potential to shape the evolution of female mating preferences.

## Introduction

Male animals often express conspicuous traits that increase their probability of attracting females, but these traits can also increase their probability of attracting predators [1]–[6]. Males of many species may thus be forced to trade off the benefits of attracting mates and the risks of attracting predators. Females often prefer males with these conspicuous traits because these males provide material benefits that increase female fitness or genetic benefits that increase offspring fitness [7]. Associating with conspicuous males, however, might increase a female's risk of predation because of conspicuous males' higher probability of attracting predators. As a result, females, like males, may be forced to trade off mating benefits and predation costs. Association costs have the potential to have a powerful effect on sexual selection. Association costs may limit the expression of female preferences or favor females that select mates based on alternative, less risky traits. Because of these effects on female preferences, association costs may also change the nature of sexual selection on male traits. Few laboratory studies have shown that females might incur association costs [8], and little is known about such costs in the field.

Field crickets provide a striking example of the predation costs of male signals. Males of some species are parasitized by the parasitoid fly, *Ormia ochracea* [1], [9], [10]. These flies locate their hosts by orienting to male song and then deposit larvae on and around males [1]. Larvae landing around males will latch onto anything moving by them [1], and in a related parasitoid, *Homotrixia alleni*, the larvae can live for up to two hours outside of a host [11]. Thus, in addition to being directly parasitized, crickets are also at risk of becoming parasitized by previously deposited larvae. Once the larvae contact a cricket, they enter the cricket where they feed and grow. The larvae emerge seven to twelve days later to pupate, and the cricket dies shortly thereafter [1], [9], [10]. Previous studies have shown that the flies preferentially orient to the same song types that female crickets prefer [12]–[15]. As a result, males that produce song types preferred by females may have a higher risk of fly parasitism [16].

Although female crickets do not sing, they are occasionally parasitized [9], [17]. Nothing is known about the context of female parasitism, but it presumably occurs when females are in association with singing males. In many field crickets, males sing from just outside the entrance to a burrow [18]. When a female approaches a singing male, she might pick up previously deposited larvae from the ground. In addition, once the female is near the male, the two directly interact for a short time while the male produces both calling and courtship songs [18]. During this time, the female might be indirectly parasitized, picking up previously deposited larvae from the ground or from the male, or be directly parasitized by a recently attracted fly. If a female decides to mate, the pair then retreats into the male's burrow where the risk of fly parasitism is likely much lower.

There is substantial evidence that being parasitized is costly for males. First, parasitized crickets die within seven to twelve days of being parasitized [1], [9], [10]. Males typically live for two to four weeks as adults in the field [19], [20], so being parasitized may often significantly reduce a male's lifespan. Second, male reproduction while still alive can be severely reduced: parasitized males sing less [21] and would therefore attract fewer mates, show reduced courtship activity [22], and have reduced reproductive effort [23]. As a result of these types of effects, Lehmann and Lehmann [24] calculated that male bushcrickets, *Poecilimon mariannae*, parasitized by *Therobia leonidei* lost 42% of their potential lifetime reproductive success compared to unparasitized males. Being parasitized is also likely to be costly for females. Like males, parasitized females have a reduced lifespan. In addition, female egg laying precipitously declines within five days of being parasitized in several species [22]. The costs of being parasitized are likely to be very high for younger males and females that are just beginning to reproduce, but even older individuals may incur costs.

In the variable field cricket, *Gryllus lineaticeps*, males that produce higher chirp rates are more likely to attract both conspecific females and parasitoid flies [12], [13]. Females receive fecundity benefits from mating with males with higher chirp rates [25], but because these males are more likely to attract parasitoid flies, females may not only risk fly parasitism when in association with males, but also may incur a greater risk when in association with preferred males. We used a field experiment to test the hypothesis that females in association with higher chirp rate song incur a higher risk of fly parasitism. We then assessed the parasitism rate of female crickets in the field and used a laboratory infection study to assess whether our estimate of female parasitism rate was biased by differences in the activity patterns of parasitized and unparasitized females.

## Methods

### Parasitism Risk Experiment

All *Gryllus lineaticeps* used in this experiment were third- or fourth-generation lab-reared offspring of field-collected females from Rancho Sierra Vista, Santa Monica Mountains National Recreation Area near Thousand Oaks, California, USA. This population is known to be parasitized by *Ormia ochracea* [13]. Matings between individuals of known ancestry were arranged to minimize inbreeding in our colony. Full sibling families were reared in 25×15×17 cm clear plastic containers that were outfitted with egg carton shelters, a paper towel substrate, vermiculite containers for oviposition, water vials with cotton plugs and ad libitum Purina Cat Chow^©^. At the penultimate stadium, individuals were moved to 15×8×11 cm clear plastic individual containers that were outfitted with shelter, substrate, water and food. In the laboratory in Lincoln, Nebraska, USA, crickets were maintained on a 14∶10 hour light:dark cycle at 23±2°C.

To test whether crickets in association with high chirp rate song have a greater parasitism risk than those in association with low chirp rate song, lab-reared crickets were transported to the field site, Rancho Sierra Vista, where we placed male-female pairs in cages above speakers broadcasting either high or low chirp rate *G. lineaticeps* calling song. The synthetic songs we used are described in Wagner and Basolo [26]. In brief, a natural pulse was digitized and used to create a chirp that contained eight pulses (chirp duration  = 120 ms). This chirp was then used to create a high chirp rate song (4.2 chirps/s) and a low chirp rate song (1.8 chirps/s), each of which was recorded to a compact disc. In the field, Pyramid MDC-6 waterproof speakers (13.35 cm diameter) were buried flush with the ground and oriented with the speaker cone facing upward. We placed cylindrical cages (15.2 cm diameter, 10.2 cm height) on top of the speakers. The cages were constructed of size five 24-gauge galvanized wire mesh; the openings in the mesh were large enough for flies to pass through but small enough to contain the crickets. The bottom of the cage was covered with speaker grill cloth to prevent parasitoid fly larvae from falling through onto the speaker. We set up ten of these cages on top of speakers; they were evenly spaced 5 m apart in two rows of five. Song was broadcast through the speakers using Coby CX-CD567 and Coby CX-CD587 weather-resistant personal compact disc players and Sonic Impact Technologies 5066 portable 15 W class-T amplifiers. For each pair of speakers, one broadcast the high chirp rate song and the other broadcast the low chirp rate song. The songs were switched between speakers between nights to prevent biases based on speaker location.

Eighty trials were run from 11–21 August 2007 in the field at Rancho Sierra Vista. All crickets used in the experiment were between 5 and 21 days post adult eclosion, lab-reared, and housed away from parasitoid flies. Thus, all crickets were known to be unparasitized at the start of the experiment. Depending upon the number of crickets of the correct age and sex that were available, we set up between six and ten cages per night. Prior to sunset, we placed a female and a muted male in each of the cages positioned above the speakers; we muted males by sealing their forewings with beeswax so that we could control chirp rate. Song was then broadcast for 30 minutes, beginning at sunset (between 2040–2050), at an amplitude of 90–92 dB SPL (re: 20 µPa) measured 35 cm above the speaker. The cages were checked for parasitoid flies 10, 20 and 30 minutes following the start of a trial using a headlamp. The song amplitudes of males are typically 67–79 dB SPL (re: 20 µPa) at 30 cm (Wagner unpublished data), and the male-female pair would only spend a few minutes together above ground while the male is singing. However, in order to complete the experiment in a practical amount of time, we purposely exaggerated the absolute parasitism risk by forcing the crickets to associate with a high amplitude song (to attract a sufficient number of flies) for a longer period (to allow sufficient time for parasitism to occur). While absolute parasitism rates for crickets in the experiment were unnaturally high, we were interested in the relative difference in risk for crickets in association with the two chirp rates. We discuss the potential consequences of this experimental design choice in the discussion.

After a trial was finished, the crickets were returned to their individual containers and monitored for parasitoid pupae for 15 days (the emergence range for *O. ochracea* pupae from *G. lineaticeps* for this experiment was 8–12 days: X±SE = 9.7±0.1 days, *N* = 82 crickets). Any cricket that died before 15 days was dissected to determine its parasitism status. Crickets remaining alive at the end of monitoring were frozen and later examined for parasitoid larvae by dissection to ensure that 15 days of monitoring was a sufficient criterion for detecting parasitism; none of these dissected crickets were parasitized. Two males escaped the cage during a trial and two males were lost before their parasitism status could be determined; data from those males were excluded, resulting in 39 high chirp rate males and 37 low chirp rate males. Females paired with the males that escaped during a trial were not included in the analysis because the absence of the male may have changed their risk of parasitism; however, females paired with males that were lost subsequent to the completion of a trial were included in the analysis, resulting in 40 high chirp rate females and 38 low chirp rate females.

### Parasitism Rates

We collected male and female *G. lineaticeps* from Rancho Sierra Vista to determine parasitism rates in the field. All crickets were collected by visually searching with a headlamp in areas with low or no vegetation (crickets are difficult to observe and collect in vegetation). In order to separate crickets collected before and during the period when flies were active, we checked for fly activity each night by observing whether flies oriented to male song. We did this by broadcasting synthetic male song from compact disc played on either Coby CX-CD567, Coby CX-CD587 or Sony CD Walkman D-EJ011 personal compact disc players and Saul Mineroff SME-AFS Portable Field Speakers at 80–90 dB SPL (re: 20 µPa) at 30 cm from speaker. In 2007, we began sampling for flies on 15 July and flies were observed at that first broadcast. In 2008, we began sampling for flies on 10 July and sampled a minimum of twice per week; flies were first observed on 15 August and did not reach appreciable numbers (greater than two per broadcast) until 30 August.

In 2007, male and female crickets were collected from 15 July to 22 August after the parasitoid flies had already become active. In 2008, female crickets were collected from 10 July to 9 August, before the flies became active, and from 15 August to 14 September, after the flies became active. No males were collected in 2008 because we were interested in focusing on female parasitism rates. In both years, crickets were collected sporadically within each time period, with the average time between collections being two days. Field collected crickets were brought to an indoor space away from flies and housed in individual plastic containers with shelter, substrate, water and food. We checked the containers daily for the presence of parasitoid pupae for a minimum of 15 days post collection. If a cricket died before 15 days without the appearance of pupae, it was dissected to determine parasitism status.

### Activity Patterns Experiment

Differences in the activity patterns of parasitized and unparasitized females might have biased our female parasitism estimates (e.g., parasitized females might spend more or less time exposed than unparasitized females). In order to assess the importance of such a bias, we examined the activity of parasitized and unparasitized female *G. lineaticeps* in an arena in the laboratory. The crickets used in this experiment were second-generation lab-reared offspring from field-caught females from Rancho Sierra Vista. To produce parasitized crickets, we transported gravid *O. ochracea* females from Rancho Sierra Vista to the laboratory in Lincoln, Nebraska. Six or fewer flies were housed in each clear plastic container (25×15×17 cm). Each container had shredded paper towel for substrate, a dish with sugar cubes and cotton that was wetted with sugar water, and another dish with natural applesauce. We then hand-infected some females by depositing larvae on the soft tissue in the space between their pronotum and wings using a dissecting probe; we attempted to deposit two larvae per cricket, but there was some variation in the number of larvae that emerged from the experimentally infected females (X±SE = 2.1±0.3 larvae, *N* = 10 crickets). The unparasitized females were sham-infected by handling them in the same manner as the hand-infected females, except we used a clean probe instead of one with larvae on it. The females were housed in individual containers with shelter, substrate, water and food in an acoustically isolated room on a 14∶10 hour light:dark cycle at 23±2°C.

Female activity patterns were assessed in a 3.65×1.2×0.65 m plywood arena. The inner walls of the arena were covered with black plastic to prevent females from climbing out of the arena, and the substrate consisted of a thin layer of sand. Twelve egg carton shelters (10×10 cm) were placed in two rows of six inside the arena; the two rows were 50 cm apart and the shelters in each row were 45 cm apart. Small plastic Petri dishes, with three pieces of cat food in each, were placed equidistant between adjacent shelters in each row to encourage the female crickets to leave the shelters and forage, as they would naturally have to leave shelter to find food. Three clip-on desk lamps with red bulbs illuminated the arena.

All crickets were tested two and six days post infection (or sham-infection). Previous work indicated that parasitism does not affect behavior or reproduction until three to five days post infection [22], so we choose a time earlier in infection where the parasitoid should have less of an effect on the host, and a time later in infection where the parasitoid should have more of an effect. Six days was chosen as the later day in order to represent all of the infected crickets as some crickets die as early as seven to eight days after being parasitized and thus would not have been able to participate in the experiment. Prior to the first test at two days, each cricket was marked using a unique combination of colored dots of correction fluid placed on the dorsal surface of the thorax. Three parasitized and three unparasitized females were tested in each trial. The six females were placed in the arena with the fluorescent room lights on for 10–12 hours prior to the start of observations. No song was broadcast during this period of simulated daylight. The room lights were then turned off and song was broadcast to simulate nighttime conditions. The high chirp rate song used to assess the effect of chirp rate on parasitism risk was broadcast at 60 dB SPL (re: 20 µPa) at 50 cm from speakers located on the ground outside each of the narrow ends of the arena. The song was broadcast from compact disc on a Sony CD Walkman D-EJ011 personal compact disc player connected to a Sonic Impact Technologies 5065 Gen2 portable 15 W class-T digital amplifier and Pyramid MDC-6 waterproof speakers (13.35 cm diameter). The broadcasts were designed to provide incentives for the female crickets to move around in the arena to search for singing males, as would occur under natural conditions. The crickets were acclimated to these nighttime conditions for one half hour before beginning the three-hour observation period. During this three-hour period, the location of each cricket was noted every 10 minutes (in the open or hiding beneath a shelter) by spot-checking with a headlamp (this was necessary to observe the unique markings on the thoraxes of the females). Each trial thus yielded 19 samples of female activity (beneath a shelter or not beneath a shelter).

A total of 12 parasitized and 12 unparasitized females were tested two and six days following infection/sham-infection between 27 September and 6 October 2008. Two of the infected females, however, did not yield parasitoid pupae. Because we could not determine parasitism status until parasitoid pupae emerged, those two crickets were run in the experiment, but they were not included in the analysis. The resulting sample size was thus 10 parasitized and 12 unparasitized females.

## Results

### Parasitism Risk Experiment

**Figure 1:**
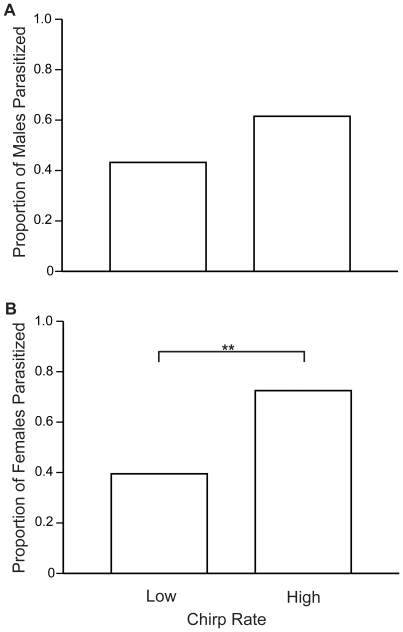
Proportion of crickets parasitized in the low and high chirp rate treatments. Male crickets (A) in the high chirp rate treatment tended to be parasitized more than males in the low chirp rate treatment. Female crickets (B) in the high chirp rate treatment were nearly twice as likely to be parasitized than females in the low chirp rate treatment. Significant differences are indicated by asterisks (** P<0.01).

Parasitoid flies were more likely to be observed in the high chirp rate (HCR) cages than in the low chirp rate (LCR) cages (HCR: 35/40, LCR: 24/38; Fisher's exact test: P = 0.017). Because flies were more likely to be attracted to the higher chirp rate song, cages in the high chirp rate treatment were more likely to contain at least one parasitized cricket than cages in the low chirp rate treatment (HCR: 34/39, LCR: 20/37; Fisher's exact test: P = 0.002). There was a tendency for males in the high chirp rate treatment to be parasitized more frequently than males in the low chirp rate treatment (HCR: 24/39, LCR: 16/37; Fisher's exact test: P = 0.168, Fig. 1A). Females in the high chirp rate treatment, however, were significantly more likely to be parasitized than females in the low chirp rate treatment (HCR: 29/40, LCR: 15/38; Fisher's exact test: P = 0.006, Fig. 1B); the parasitism risk for females in the high chirp rate treatment was 1.8 times greater than that for females in the low chirp rate treatment.

### Parasitism Rates

In the 2007 collection, which occurred during an unknown period of time after the start of fly activity, approximately 1% of female crickets collected were parasitized (1 of 104) and 59.1% of male crickets collected were parasitized (13 of 22). The disparity between the number of females and males collected was probably due to the lower likelihood of encountering males using visual search methods; males remain near their burrows during nighttime hours whereas females move around actively searching for males. In the 2008 collection, no females were parasitized before the flies were observed (0 of 50), while 6.1% of females were parasitized after the flies were observed (3 of 49). No males were collected in 2008.

### Activity Patterns Experiment

**Figure 2:**
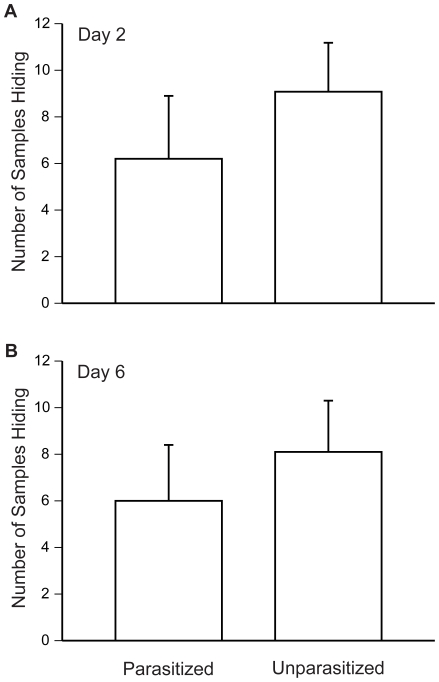
Number of sampling periods in which female crickets were hiding. We found no difference in the number of sampling periods in which parasitized and unparasitized female crickets were hidden under a shelter on either day 2 (A) or day 6 (B) in the activity patterns experiment. Means plus one standard error are shown.

Parasitized and unparasitized female crickets did not differ in the number of samples in which they were hidden under shelters, either two days following parasitism (Mann-Whitney *U* test: z_20_ = 1.051, P = 0.293, Fig. 2A) or six days following parasitism (Mann-Whitney *U* test: z_20_ = 0.840, P = 0.401, Fig. 2B). Furthermore, neither parasitized females (Wilcoxon matched-pairs signed-rank test: z_8_ = 0.255, P = 0.799) nor unparasitized females (Wilcoxon matched-pairs signed-rank test: z_10_ = 0.237, P = 0.813) showed changes in their shelter use from day two to day six.

## Discussion

In the variable field cricket, *Gryllus lineaticeps*, females from at least some populations prefer males that produce higher chirp rate song [12], and males with higher chirp rates appear to transfer seminal fluid products to females that enhance female fecundity [25]. Our results, however, suggest that to obtain these benefits, females in populations parasitized by *Ormia ochracea* may have to incur a greater risk of fly parasitism. In our field experiment, females in association with high chirp rate song were 1.8 times more likely to be parasitized than females in association with low chirp rate song. This greater risk is at least partially because higher chirp rates are more likely to attract flies, as was found in this and other studies [12], [13]. Because the inevitable result of fly parasitism is death, the cost for females is potentially quite severe, particularly for young females that may lose a substantial proportion of their reproductive lifespan if they are parasitized. This cost is magnified by the fact that female egg laying drops sharply between three and five days post infection [22]. Field crickets typically live for two to four weeks as adults in the field [19], [20], and rather than periodically laying discrete clutches of eggs, females lay a small number of eggs each day they remain alive. If a female is parasitized at a young age, her reproductive lifespan will be reduced from a few weeks to a few days, which should substantially reduce her lifetime reproductive success. Because of the cost of fly parasitism, and because of the higher risk that appears to result from being near high chirp rate song, the evolution of female song preferences in this species may thus be affected not only by the benefits of mating with high chirp rate males, but also by the risk of fly parasitism that results from associating with them.

Surprisingly, males in the high chirp rate treatment were not parasitized significantly more often than males in the low chirp rate treatment, despite the fact that the high chirp rate cages were significantly more likely to attract flies. There are several possible explanations for this puzzling result. While the difference was not statistically significant, there was a tendency for high chirp rate males to be parasitized more frequently than low chirp rate males. A larger sample size might have allowed us to detect a difference. Because females were present for the entire duration of fly exposure, the overall male parasitism rate may have been lower making it more difficult for us to detect relative differences between the chirp rate treatments. For instance, it is possible that the flies use cues other than sound to choose hosts once the general location of the host is established; the females, which are on average larger than males, might be easier or more profitable targets for the flies. Additionally, females might have been more active in the cages, resulting in a higher encounter rate with flies and/or larvae deposited on the substrate. And finally, males are likely to have evolved more effective anti-parasitoid tactics than females, as males are likely under stronger selection from fly parasitism.

Our experimental design purposely exaggerated the absolute risk of fly parasitism for females so that we could examine differences in relative risk using a practical number of replicates. The primary exaggerations were broadcasting male song at a high amplitude and forcing the females to remain in association with males while above ground for an extended period of time. For these reasons, the absolute parasitism risk for females and males is certainly much lower than our experiment would suggest. It is also possible that these methodological choices biased our estimates of the relative risk of associating with high and low chirp rate song. While it seems unlikely that the use of high amplitude song could cause a difference in relative risk that is otherwise not present, the real relative risk could be lower or higher than we found in our experiment depending upon whether the flies show either a lesser or greater chirp rate discrimination at high amplitudes. Ramsauer and Robert [27] found that the flies would respond to simulated *G. rubens* song with carrier frequencies not naturally present in their songs when presented at high amplitudes, suggesting that the flies may actually be less discriminating at high amplitudes. Whether a long duration of association could bias estimates of relative risk depends, in part, on whether females that approach high and low chirp rate males spend different amounts of time above ground before entering the male's burrow where the female's risk is likely much reduced. If females that approach high chirp rate males and females that approach low chirp rate males spend similar amounts of time above ground before entering the burrow, the natural difference in relative risk should be similar. If, however, females take longer to enter the burrows of low chirp rate males, the natural difference in relative risk may be less than our results suggest; taking longer to enter may increase the risk that a fly will arrive before the female enters the burrow and may also increase the risk the female will pick up previously deposited larvae. Such a difference in behavior should be disfavored by selection. If there is a risk of fly parasitism, females should only approach males with which they are interested in mating, and they should quickly enter the male's burrow. Once in the burrow, they can assess non-calling song traits (e.g., courtship song, tactile signals and any chemical signals) with less risk. In addition to exaggerating some conditions, we chose to base all song characteristics (chirp duration, dominant frequency, etc.) except chirp rate on the average value for our population for both the high and low chirp rate stimuli. This could create issues for generalizing the results; for instance, perhaps the flies would respond differently if we used long chirp durations instead of average chirp durations.

In field samples collected during periods of fly activity, 1 and 6% of the females were parasitized. In related species attacked by *O. ochracea*, Walker and Wineriter [9] found that approximately 10% of *G. rubens* and 10% of *G. firmus* females collected by systematic search were parasitized, and Adamo et al. [17] found that 3.2% of *G. integer* females that responded to male song broadcasts were parasitized. These studies and ours likely underestimate the actual parasitism rate for females. First, females do not become sexually mature until approximately seven days following their final molt, and as a result, some of the females collected might not have been sexually mature, and thus might not have had opportunities to become parasitized. Second, the unparasitized females that were sexually mature likely had a non-zero probability of later becoming parasitized had they not been collected. And third, it is possible that estimates of female parasitism rates could be biased by unequal probabilities of encountering parasitized and unparasitized females. This could occur because parasitized females die at a faster rate and thus are less likely to be encountered and/or because parasitized and unparasitized females differ in their activity patterns. For example, once parasitized, females might spend less time moving around above ground in search of food or mates, which could make them less likely to be collected using a visual search method. In our activity patterns experiment, we found that parasitized females did not hide more often than unparasitized females, suggesting that differences in female activity probably did not substantially bias our parasitism rate estimates in *G. lineaticeps.* We did not, however, examine female activity in the later stages of parasitism (>6 days post infection), which could affect the probability of parasitized females being represented in field samples as activity could change very late in parasitism.

Fly parasitism appears to have affected the evolution of male mating behavior in a number of species [10], [28]–[32]. Whether the risk of fly parasitism for females is, or has been, sufficiently high enough to affect the evolution of female mating behavior is not known. Studies of a variety of organisms suggest that directional natural selection is typically weak [33], [34]. Even small effects on fitness, however, can result in large evolutionary changes given the cumulative effect of selection over multiple generations. Given the relatively large difference in female risk when in association with high and low chirp rate males in *G. lineaticeps*, selection may be sufficiently strong to favor female behaviors that reduce the risk of fly parasitism, such as weaker preferences for high chirp rate males. In addition, the relatively low parasitism rates of females in nature might be a consequence of effective anti-parasitoid tactics that have already evolved, such as mating during times when the flies are less active [35], mating less frequently and/or choosing less risky males. For instance, in a single parasitized population of *G. rubens*, Velez and Brockman [36] found that autumn females, which experience fly parasitism, were less responsive to male song than spring females, which do not experience fly parasitism. However, comparative studies of parasitized and unparasitized populations will be necessary to determine if the risk of fly parasitism has affected the evolution of female mating behavior, and whether the greater risk of associating with high chirp rate males has affected the evolution of female mating preferences.

Little is known about the predation risk that female animals incur from associating with males with more and less preferred traits, despite the importance of such costs for the evolution of female mating preferences. Choosy females, however, may risk predation in any species where preferred males are more conspicuous and likely to attract predators. Mate choice may thus often require a compromise between the benefits of mating with more preferred males and the lower risk of predation that results from mating with less preferred males. Guppies, *Poecilia reticulata*, are one of the only animals for which data are available on female association costs and the evolutionary consequences of these costs. Controlled laboratory experiments suggest that female guppies have a greater risk of predation by a piscivorous cichlid when near more colorful males [8], and female guppies from populations with a higher risk of predation have weaker preferences for conspicuously colored males [37]. Indirect evidence suggests that predation has had important effects on the evolution of female preferences in a variety of animals. For example, females in many species change their preferences when the perceived risk of predation is high [38]–[43]. Though these studies do not directly demonstrate the costs to females of being near conspicuous males, their results are consistent with an effect of these association costs on the evolution of female preferences. Because costs of female preferences can have profound effects on the nature and direction of sexual selection, more studies are needed to examine the existence of these costs in other taxa, as well as the evolutionary consequences of these costs in this species and other taxa.
